# The Effect of Integrative Neuromuscular Training on Enhancing Athletic Performance: A Systematic Review and Meta-Analysis

**DOI:** 10.3390/life15081183

**Published:** 2025-07-25

**Authors:** Baili Chen, Lin Deng, Yuhang Liu, Xiaojing Deng, Xiaoyi Yuan

**Affiliations:** 1 College of Education, Beijing Sports University, Beijing 100084, China; 2 State General Administration of Sport Key Laboratory of Sports Training, Beijing 100084, China

**Keywords:** integrative neuromuscular training, athletic performance, meta-analysis

## Abstract

**Purpose**: Integrated neuromuscular training (INT) is a multidimensional training method that integrates strength, balance, core stability, flexibility, and motor skill development. The aim of this study was to systematically evaluate the effects of INT on various physical performance indicators in athletes to provide evidence supporting the application of INT in training practices. **Method**: A systematic search was conducted in accordance with PRISMA guidelines across nine databases—including PubMed, Web of Science, EMBASE, Scopus, Cochrane Library, Ovid MEDLINE, WILEY, and Springer Nature Link—from inception to 26 March 2025 to identify randomized controlled trials (RCTs) examining the effects of INT on athletic performance. **Result**: A total of 19 randomized controlled trials were included, comprising 783 participants aged 11–25 years. The meta-analysis results indicated that INT significantly improved jump performance (SMD = 0.26, 95% CI [0.15, 0.37], *p* < 0.001, I^2^ = 75%), sprint performance (SMD = −0.76, 95% CI [−0.93, −0.58], *p* < 0.001, I^2^ = 76%), balance performance (SMD = 0.23, 95% CI [0.14, 0.31], *p* < 0.001, I^2^ = 78%), and agility performance (SMD = −0.72, 95% CI [−1.23, −0.21], *p* < 0.05, I^2^ = 74%). Sensitivity analyses revealed no substantial changes in jump, sprint, agility, or balance performance outcomes. **Conclusions**: INT was found to significantly improve sprint, jump, balance, and agility performance in athletes. Analysis of the included training protocols suggested that improvements in each performance indicator required emphasis on specific training components. Moreover, greater improvements in sprint and balance performance were observed in female athletes compared to their male counterparts. Subgroup analysis revealed significant differences in training effects across populations, with female athletes showing superior improvements in sprint and balance performance following INT interventions. Additionally, interventions lasting fewer than eight weeks, with training sessions shorter than 30 min and frequencies of more than three times per week, were associated with more pronounced effects.

## 1. Introduction

Athletic performance is a multidimensional construct comprising physical fitness, psychological attributes, technical skills, and tactical awareness and is primarily reflected in the athlete’s competitive level demonstrated during training or competition [1]. To enhance athletic performance, sports scientists have systematically developed diverse training programs tailored to various athletic abilities. For instance, lower-limb explosive strength can be enhanced through stretch-shortening cycle (SSC) training [2,3,4], functional training to improve integrated movement capacity [5,6], and eccentric overload training [7,8,9]. In addition, aerobic endurance can be improved by using altitude training strategies [10,11].

Integrative neuromuscular training (INT) is a conceptual and multidimensional training model. It combines fundamental and targeted training components, including resistance training, dynamic stability training, core strength training, plyometric training, and agility training, addressing both health- and skill-related physical fitness elements [12,13]. Compared to traditional neuromuscular training, INT emphasizes an integrated training model that concurrently enhances muscular strength and motor skills [14,15]. Moreover, INT has been shown to effectively improve sprint and change-of-direction performance [16] as well as reduce the risk of sports-related injuries [16]. Traditional resistance training (RT) refers to a method in which skeletal muscles actively contract to overcome external resistance. This type of training is typically performed with periodic and progressive loading to stimulate muscle hypertrophy and enhance muscular strength [17]. In contrast, the physiological mechanism through which INT improves athletic performance and reduces injuries involves stimulating the sensorimotor system and integrating complex movement information into the central nervous system to achieve enhanced motor control [12]. This training approach aligns with the physiological mechanisms of multi-system coordination during movement and better reflects the actual demands of human motor performance.

Recent studies have demonstrated that implementing INT prior to sport-specific training yields greater benefits compared to post-training implementation [18] and that performance improvements can be sustained for a period following the intervention [19]. In several randomized controlled trials (RCTs), although specific INT protocols were applied, inconsistent results have been reported regarding improvements in physical performance indicators. For example, conflicting findings have been observed in sprinting performance [20,21,22] and change of direction (COD) ability [23]. Moreover, further investigation is warranted into the effects of training duration, frequency, and session length on lower-limb explosive strength, balance, and agility as well as the differential impact of INT across various sports, age groups, and sexes and whether current assessment tools can accurately evaluate athletic performance.

This systematic review and meta-analysis aims to evaluate the effects of integrative neuromuscular training (INT) on athletes’ performance in jump, sprint, agility, and balance tasks. The study also explores the differential effects of training frequency, duration, and total intervention period to provide practical implications for coaches and athletes.

## 2. Materials and Methods

This study was conducted in strict accordance with the Preferred Reporting Items for Systematic Reviews and Meta-Analyses (PRISMA) guidelines. The literature search was conducted for randomized controlled trials (RCTs), published from database inception to 26 March 2025, that investigated the effects of integrative neuromuscular training on athletic performance. Study selection and data extraction were completed from March to April 2025. Quality assessment and meta-analysis were carried out in early May, and manuscript writing and revisions were completed in late May prior to submission. The review protocol was registered in PROSPERO prior to the literature search (registration number: CRD420251018577). Risk of bias was assessed using Review Manager 5.3, and a random-effects model was employed to calculate effect sizes based on group means and baseline standard deviations (pre-SD). Results were presented as standardized mean differences (SMDs) with 95% confidence intervals (CIs).

### 2.1. Search Strategy

This review followed the PRISMA guidelines, and the search period was set from database inception to 26 March 2025, targeting randomized controlled trials (RCTs) investigating the effects of integrative neuromuscular training on athletic performance. Two independent reviewers conducted a blinded search of the PubMed, Web of Science, EMBASE, Scopus, Google Scholar, Cochrane Library, MEDLINE, and Science Citation Index databases. A Boolean search was performed using the following keywords: “integrative neuromuscular training” OR “integrative neuromuscular exercise” OR “integrative neuromotor training” OR “integrative neuromotor exercise” OR “neuromotor training” OR “neuromotor exercise” OR “neuromuscular training” OR “neuromuscular exercise” AND “performance*” OR “sprint” OR “agility” OR “balance”. Citation tracking was also employed to ensure comprehensive coverage. A total of 8364 English-language articles were initially identified. Any discrepancies between the two reviewers were resolved through discussion or adjudication by a third reviewer.

### 2.2. Inclusion/Exclusion Criteria

#### 2.2.1. Inclusion Criteria

Based on the PICOS criteria of the Cochrane systematic review framework, the inclusion criteria were as follows in Table 1: (1) Population: athletes (either professional or amateur) with at least 12 months of regular training experience, engaging in training at least once per week, and aged 11 years or older; (2) Intervention: studies employing integrative neuromuscular training (INT) or neuromuscular training (NT); (3) Comparison: the intervention group received any form of integrative neuromuscular training, with an intervention duration of at least 4 weeks and each session lasting no less than 15 min; (4) Outcomes: the outcome measures included physical performance indicators, such as speed, agility, and balance; (5) Study Design: only randomized controlled trials (RCTs) with a clearly defined and complete intervention protocol were included.

#### 2.2.2. Exclusion Criteria

The exclusion criteria were as follows: (1) non-athletes or athletes with existing injuries; (2) studies with incomplete data, reporting only partial pre- and post-intervention outcomes; (3) secondary reviews or studies not based on original experimental data; (4) studies lacking clear descriptions of experimental design, procedures, data collection, or statistical analysis; (5) conference abstracts, reviews, dissertations, and qualitative studies; (6) non-English publications.

### 2.3. Screening of Literature and Data Extraction

**Figure 1:**
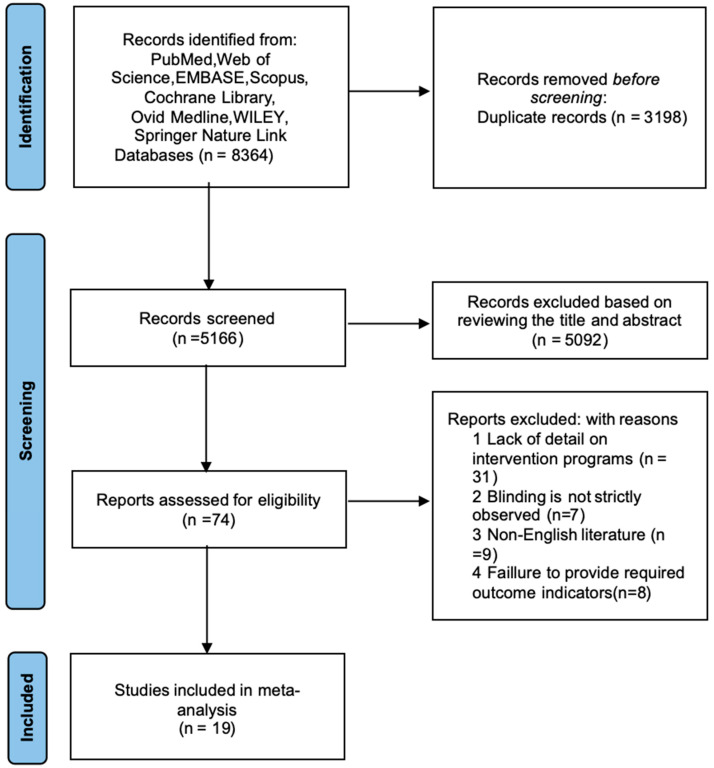
Literature screening.

This systematic review strictly followed the PRISMA guidelines (Figure 1), employing a double-blind screening approach conducted by two independent reviewers (B.C. and L.D.). EndNote 20 was used to manage and screen the retrieved articles. Based on the inclusion and exclusion criteria, both reviewers independently conducted an initial screening (titles and abstracts) and a full-text screening. Studies not meeting the criteria were excluded, and those meeting the criteria were retained. Any disagreements during the screening process were resolved through discussion with a third reviewer (H.L.) to ensure consistency and objectivity in the final selection.

Upon final inclusion, the two reviewers independently extracted data using a custom-designed standardized data extraction form. Key data included study characteristics (first author and year of publication), participant characteristics (gender and age), intervention details (type, duration, frequency, and intensity), and post-intervention outcomes related to athletic performance, such as speed, power, agility, coordination, and balance.

In cases where information was incomplete, unclear, or only presented in graphical form, the corresponding authors were contacted for additional data when necessary. This approach aligns with best practices for missing data management in systematic reviews and meta-analyses, enhancing data completeness and ensuring the accuracy and reliability of the final conclusions.

### 2.4. Risk of Bias Assessment

Two independent reviewers used Review Manager 5.3 and the Cochrane Risk of Bias assessment tool to evaluate the methodological quality of the included studies. The evaluation was conducted across seven domains: (1) random sequence generation (selection bias); (2) allocation concealment (selection bias); (3) blinding of participants and personnel (performance bias); (4) blinding of outcome assessment (detection bias); (5) completeness of outcome data (attrition bias); (6) selective reporting (reporting bias); and (7) other sources of bias. For each domain, the risk of bias was classified as “low”, “high”, or “unclear”. Discrepancies between the two reviewers were resolved through discussion with a third reviewer to reach consensus on the final judgment. This process ensured the rigor and consistency of the quality assessment and provided a reliable basis for subsequent bias analysis.

### 2.5. Data Analysis

The pooled effect sizes of integrated neuromuscular training (INT) on various performance outcomes were analyzed using Review Manager 5.4. Standardized mean differences (SMDs) with 95% confidence intervals (CIs) were calculated to represent the magnitude of effect. According to the Cochrane Handbook for Systematic Reviews of Interventions, either a fixed-effect model or a random-effects model was selected based on the degree of heterogeneity among studies. Heterogeneity was assessed using the I^2^ statistic and the *p*-value. I^2^ values of 25%, 50%, and 75% were considered to indicate low, moderate, and high heterogeneity, respectively [24]. When substantial heterogeneity was observed (I^2^ ≥ 50% and *p* < 0.05), a random-effects model was applied. Otherwise, a fixed-effect model was used.

Because the included studies used different measurement tools and units for outcome variables (e.g., sprint performance measured by 10 m or 40 m dash time; explosive power measured by standing long jump distance or vertical jump height), direct pooling of raw scores was not feasible. To address this, all outcomes were standardized. The effect sizes were therefore expressed as standardized mean differences (SMDs) with 95% CIs, allowing for comparisons across different scales.

The formula used to calculate the SMD is as follows:

$$\mathrm{SMD}\;=\;\frac{Difference\;in\;mean\;outcome\;between\;groups}{\mathrm{Standard}\;\mathrm{deviation}\;\mathrm{of}\;\mathrm{outcome}\;\mathrm{among}\;\mathrm{participants}}$$

Meta-regression was conducted to explore the potential sources and extent of heterogeneity. Subgroup analyses were performed based on categorical variables such as intervention frequency, intervention duration, and participant age to determine which factors were associated with greater improvements in athletic performance.

To assess the robustness of the meta-analysis results, sensitivity analysis was conducted using a leave-one-out approach. Each study was sequentially excluded, and the analysis was rerun to observe changes in the overall effect size. If the exclusion of any single study caused the pooled estimate to fall outside the 95% confidence interval of the overall effect, the study was considered to have a significant influence on the pooled result, suggesting potential bias.

Publication bias was assessed by visual inspection of funnel plots and statistically tested using Egger’s regression test, conducted with Stata version 18.0.

## 3. Results

### 3.1. Study Selection

A total of 8364 records were identified from nine electronic databases. After removing 3198 duplicates using EndNote 20 software, 5166 records remained for the initial screening. Based on the inclusion criteria, 5092 articles were excluded by screening titles and abstracts, including reviews, systematic reviews, meta-analyses, animal experiments, and conference abstracts. A total of 74 articles were subjected to full-text review. After reviewing the full texts, studies that did not meet the inclusion criteria were excluded. Ultimately, 19 studies met the inclusion criteria and were included in the final analysis.

### 3.2. Study Characteristics and Risk of Bias

**Figure 2:**
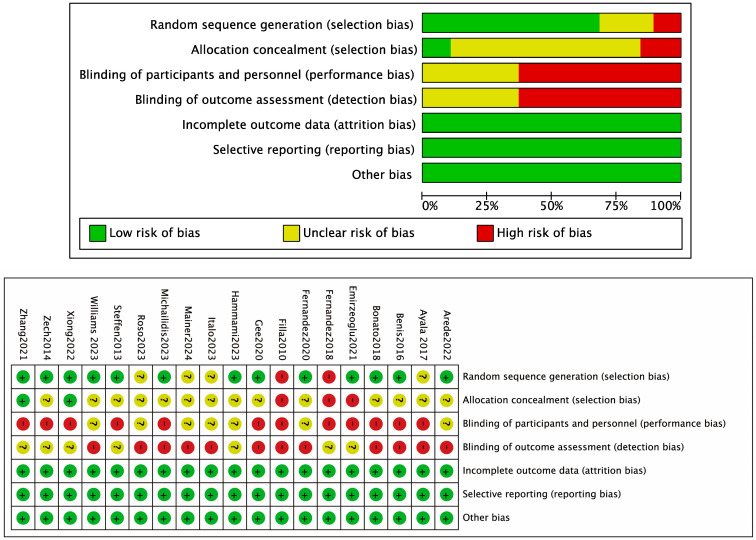
Risk Assessment of Included Studies.

**Figure 3:**
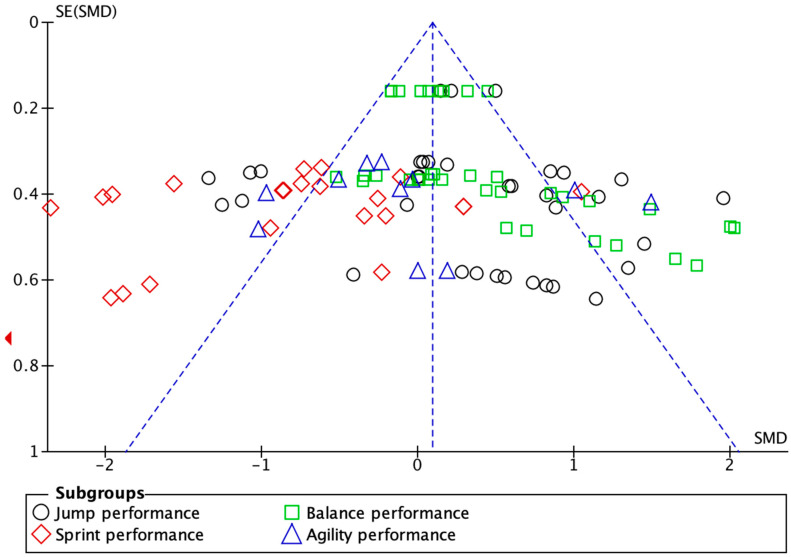
Funnel plot of the effect of INT on athletes’ performance.

A total of 19 randomized controlled trials (RCTs) were included, involving 391 participants in the intervention groups and 392 in the control groups. The basic characteristics of the included studies are summarized in Table 2. Risk of bias was generally low across the included studies, indicating a high methodological quality of the meta-analysis (Figure 2). Funnel plot inspection revealed no significant evidence of publication bias (Figure 3), suggesting that the included studies are of relatively high quality and suitable for secondary analysis.

### 3.3. Meta-Analysis Results

#### 3.3.1. Pooled Outcomes

**Figure 4:**
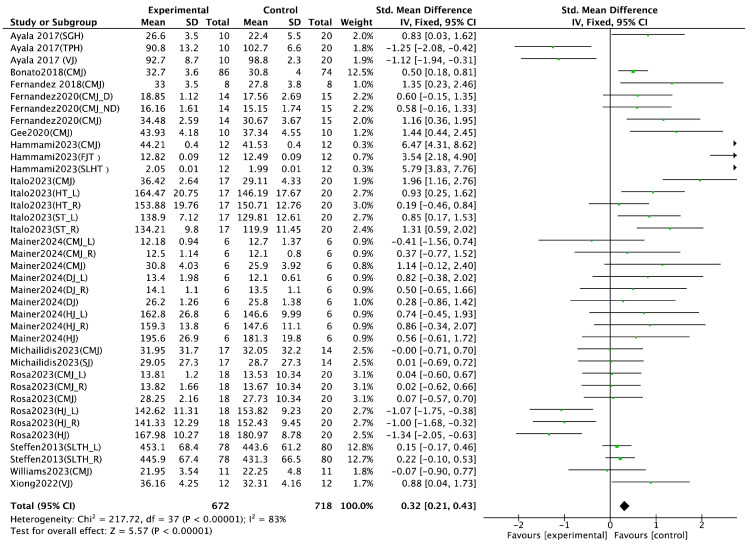
Forest plot of the effect of INT on athletes’ jump performance [18,21,22,23,24,25,26,29,31,32,34,37,39]. SGH: single hop; TPH: triple hop; FJT: five jump test; SLHT: single-leg hop test; CMJ: countermovement jump; D: dominant side; ND: non-dominant side; R: right; L: left; HT: hop test limb; ST: side test limb; DJ: drop jump; HJ: horizontal jump; VJ: vertical jump.

**Figure 5:**
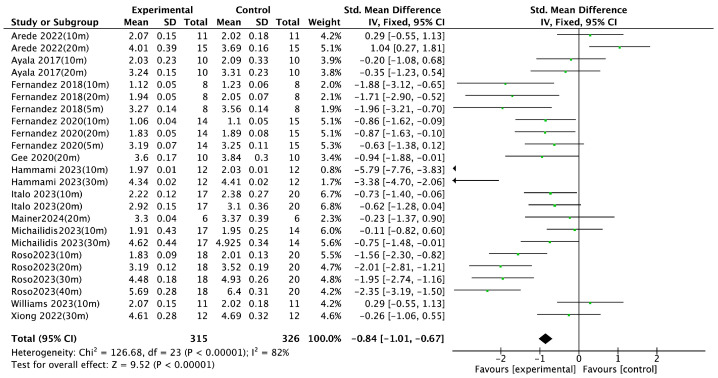
Forest plot of the effect of INT on athletes’ sprint performance [18,21,22,23,26,27,29,31,32,35,37,39]. 5 m: 5 m sprint test; 10 m: 10 m sprint test; 20 m: 20 m sprint test; 30 m: 30 m sprint test; 40 m: 40 m sprint test.

**Figure 6:**
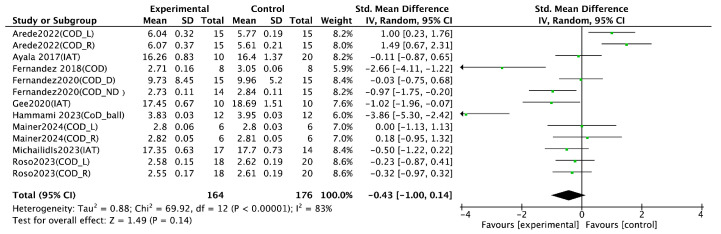
Forest plot of the effect of INT on athletes’ agility performance [18,21,23,27,31,32,35,37,39]. R: right; L: left; D: dominant side; ND: non-dominant side; COD: change of direction; IAT: Illinois agility test.

**Figure 7:**
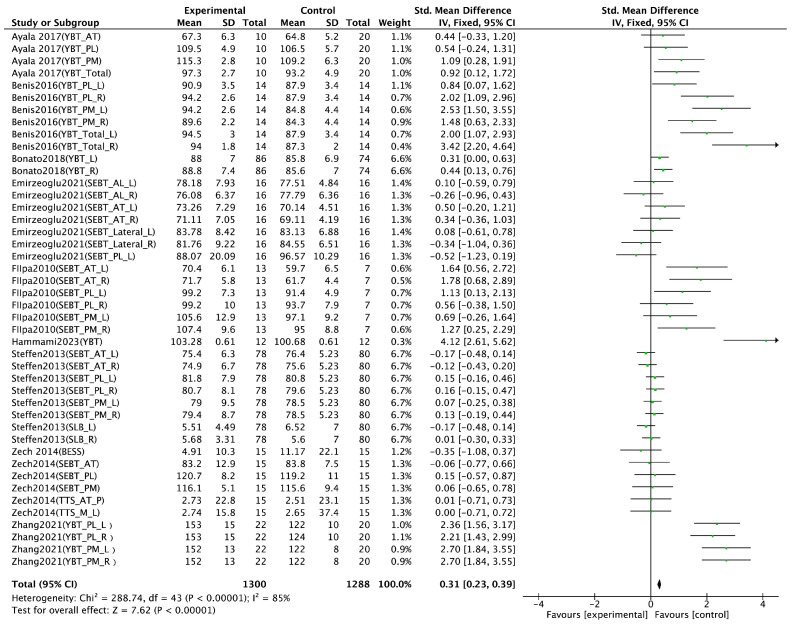
Forest plot of the effect of INT on athletes’ balance performance [21,28,30,33,34,36,37,38,39]. R: right; L: left, AT: anterior, PL: posterolateral, PM: posteromedial, SEBT: star excursion balance test, SLB: single-leg balance, BESS: balance error scoring system, TTS: time to stabilization, YBT: Y-balance test.

Jump performance, as a key indicator of lower-limb explosive strength, was analyzed based on 13 randomized controlled trials (RCTs). Various assessment methods were employed across studies, including the countermovement jump [18,21,23,25,26,29,32,35,37]; hop test [29]; side hop test [29]; drop jump [23]; horizontal jump [23,26]; and vertical jump [22,39]. The meta-analysis revealed a significant positive effect of integrated neuromuscular training (INT) on jump performance (SMD = 0.32, 95% CI [0.21, 0.43], *p* < 0.00001), with substantial heterogeneity (I^2^ = 83%) (Figure 4). Sprint performance was also examined in 13 RCTs, with all studies measuring sprint time across varying distances, including 5 M [31], 10 M [18,21,22,25,26,27,29,31,32,39], 20 M [18,23,26,27,29,31,39], 30 M [21,22,26,32], and 40 M [26]. The pooled results indicated a significant improvement in sprint performance following INT (SMD = −0.84, 95% CI [−1.01, −0.67], *p* < 0.00001), accompanied by high heterogeneity (I^2^ = 82%) (Figure 5). Agility performance was analyzed based on nine RCTs, including six studies using change of direction (COD) tests [18,21,23,26,27,31] and three using the Illinois agility test [24,29,37]. However, the meta-analysis did not find a statistically significant effect of INT on agility performance (SMD = −0.43, 95% CI [−1.00, 0.14], I^2^ = 83%, *p* = 0.14) (Figure 6). Balance performance was evaluated in nine RCTs, using various assessment tools such as the star excursion balance test [28,30,34,36], single-leg balance [36], balance error scoring system [34], time to stabilization [28], and Y-balance test [21,33,37,38,39]. The pooled analysis demonstrated a significant improvement in balance performance after INT (SMD = 0.31, 95% CI [0.23, 0.39], I^2^ = 85%, *p* < 0.00001) (Figure 7).

#### 3.3.2. Heterogeneity

The meta-analysis results demonstrated that integrative neuromuscular training (INT) was found to significantly enhance athletes’ jump, sprint, balance, and agility performances (*p* < 0.05). These findings suggest that this training modality may contribute to improved specific motor skills and overall athletic performance. Further heterogeneity tests revealed that the I^2^ values for jump, sprint, agility, and balance performances were 83%, 82%, 83%, and 85%, respectively, indicating moderate to high heterogeneity across the included studies. Therefore, sensitivity analyses were conducted to explore the potential sources of heterogeneity and improve the robustness and credibility of the findings.

#### 3.3.3. Sensitivity Analysis

**Figure 8:**
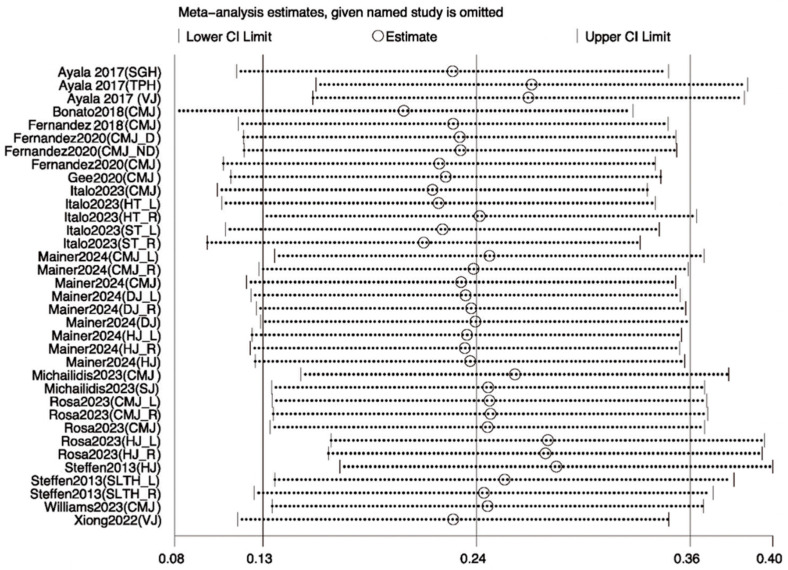
Sensitivity analysis of the effect of INT on athletes, jump performance [18,22,23,25,26,29,31,32,34,35,37,39].

**Figure 9:**
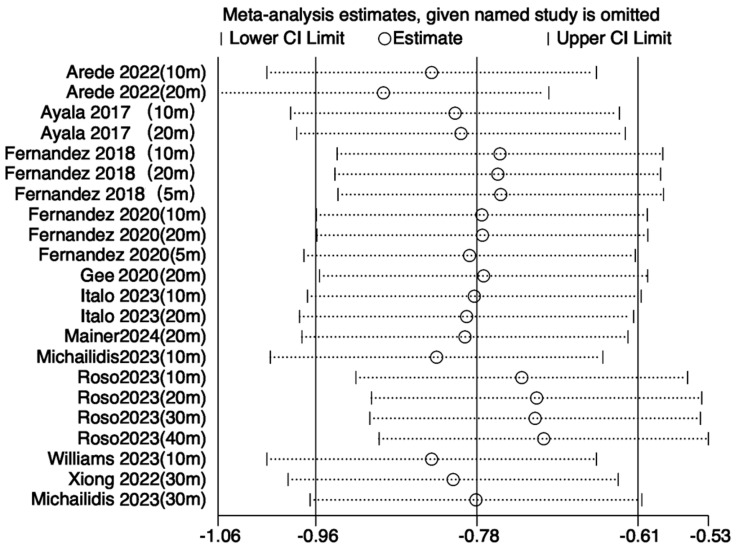
Sensitivity analysis of the effect of INT on athletes’ sprint performance [18,22,23,26,27,29,31,32,35,37,39].

**Figure 10:**
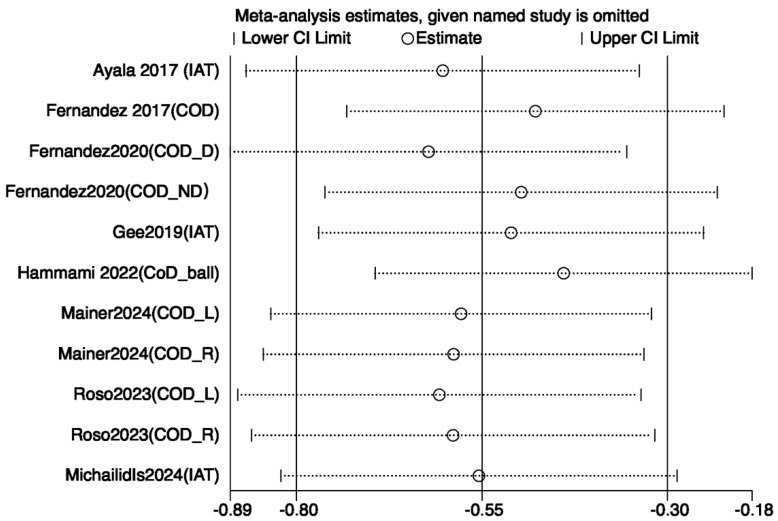
Sensitivity analysis of the effect of INT on athletes’ agility performance [18,21,23,26,31,32,35,39].

**Figure 11:**
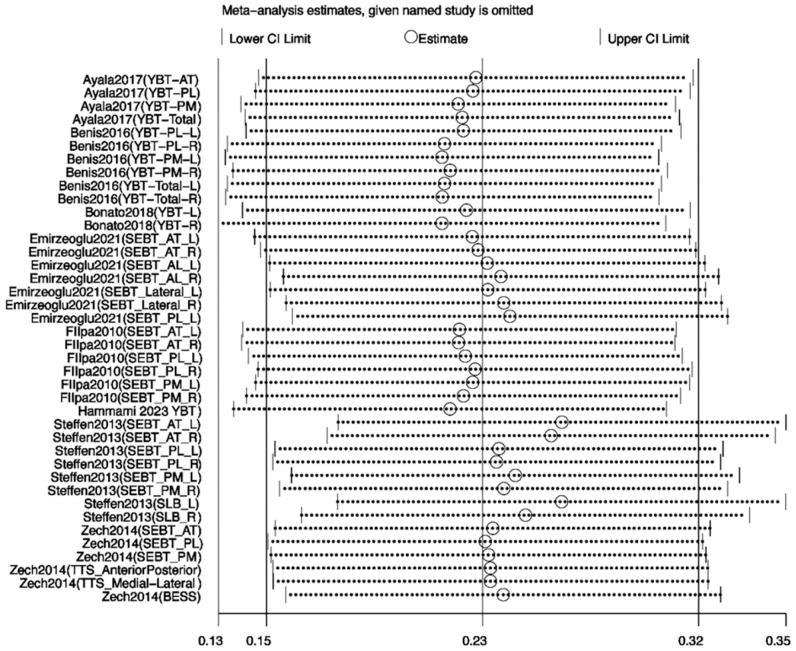
Sensitivity analysis of the effect of INT on athletes’ balance performance [21,28,30,34,36,37,38,39].

To assess the robustness of the meta-analysis results and explore the sources of heterogeneity, sensitivity analyses were performed for outcome variables with I^2^ values exceeding 50%—including jump, sprint, agility, and balance performances. Leave-one-out analyses were conducted using Review Manager 5.4, following predefined criteria such as low methodological quality, small sample size, or effect sizes deviating markedly from other studies. The remaining data were reanalyzed using STATA 18.0. For jump performance, the study by Hammami et al. [21] was considered a primary source of heterogeneity; its exclusion reduced I^2^ from 83% to 75%, with the *p*-value remaining below 0.05, indicating a stable effect (Figure 8). Similarly, for sprint performance, removing the same study reduced I^2^ from 83% to 74% with stable results (Figure 9). In the case of agility, exclusion of Arede et al. [27] decreased I^2^ from 83% to 74%, and the *p*-value dropped below 0.05 (Figure 10). For balance, exclusion of Zhang et al. [33] lowered I^2^ from 85% to 78% (Figure 11). Overall, the removal of high-heterogeneity studies did not affect the statistical significance of INT on performance outcomes, supporting the consistency and reliability of the results.

#### 3.3.4. Bias Test

Since all outcome indicators included in this study were continuous variables, Egger’s test was selected to assess the risk of publication bias. The results showed *p* > 0.05, indicating a low risk of publication bias, which supports the reliability of the meta-analysis findings.

#### 3.3.5. Subgroup Analyses

To further explore the effects of INT on jump, sprint, agility, and balance, subgroup analyses were conducted based on sex, age, and intervention parameters.

##### Subgroup of Jump Performance

Subgroup analyses revealed that the effects of INT on jump performance varied across different populations and intervention characteristics (Table 3). Specifically, INT significantly improved jump performance in athletes aged ≥15 years, while no comparable effects were observed in participants aged <15 or in mixed-age groups, suggesting that the stage of physical development may influence intervention outcomes. Moreover, male participants demonstrated more pronounced improvements compared to females or mixed-gender samples.

In terms of training parameters, interventions with ≥30 min per session and a total duration of ≤8 weeks were more effective in enhancing jump performance. A training frequency of twice per week yielded better results than ≥3 times per week. Excessively high training frequency may reduce effectiveness due to accumulated fatigue, diminished training adaptability, or suboptimal program design.

##### Subgroup of Sprint Performance

In the age-based subgroup analysis, INT interventions produced more significant improvements in sprint performance among participants aged <15 years. This may be due to age- and development-related variability in training responsiveness. In the gender-based analysis, females showed greater improvement than males, with a substantially higher effect size.

Regarding intervention duration, sessions lasting <30 min were more effective than those ≥30 min, although both showed improvements. Interventions lasting ≤8 weeks were associated with more consistent effects, indicating that shorter durations may help avoid the accumulation of neuromuscular fatigue. While a training frequency of ≥3 sessions per week resulted in the highest effect size, it was also associated with greater heterogeneity, suggesting that high-frequency training may lead to adverse adaptations in sprint performance (Table 4).

##### Subgroup of Agility Performance

Age-based subgroup analysis indicated that participants aged >15 years experienced greater improvements in agility compared to those ≤15 years. This may reflect differences in neuromuscular control and skill transfer capacity during maturation. In terms of sex-based differences, female participants exhibited significantly greater improvements than males, suggesting sex-specific responsiveness to INT in agility-related tasks.

Subgroup analysis of intervention duration showed that programs with session durations <30 min yielded more notable improvements in agility, whereas no significant changes were observed in the ≥30 min group. No consistent patterns were found in intervention duration or frequency, although training twice per week appeared more effective, indicating that excessive frequency may not yield additional benefits (Table 5).

##### Subgroup of Balance Performance

Sex-based subgroup analysis showed that females experienced more substantial improvements in balance compared to males, indicating that INT may be particularly beneficial for enhancing proprioception and postural control in female athletes. Interventions lasting <30 min per session showed higher effect sizes than those ≥30 min, although the latter still had positive effects.

With respect to training frequency, programs conducted ≥3 times per week outperformed those with only two weekly sessions, suggesting that higher frequencies may result in greater balance improvements. However, interventions lasting ≤8 weeks demonstrated the greatest effects, while those exceeding 8 weeks showed no significant improvement, indicating a potential plateau or diminishing returns with prolonged training durations (Table 6).

## 4. Discussion

Compared with conventional methods such as resistance training (RT) and physical training (PT), INT demonstrated more comprehensive training benefits. For instance, a meta-analysis of 35 studies involving 777 elite athletes revealed that RT significantly enhances sport-specific performance [40]. However, its effectiveness is significantly influenced by athletic level, sex, and performance type, indicating task- and population-specific outcomes. Another review comparing unilateral and bilateral RT found no significant difference in overall performance but noted that unilateral RT improved single-leg jump performance, while bilateral RT was more effective for bilateral strength development—further supporting the principle of training specificity [41]. In contrast, INT integrates strength, balance, coordination, and motor control in a systematic approach. This integrated nature enables INT to provide greater benefits in agility and balance—capabilities that are often insufficiently addressed by traditional RT or PT. Thus, as a multi-targeted and structured training strategy, INT can complement the limitations of traditional approaches and may offer a more holistic solution for enhancing overall athletic performance across different athlete populations.

The primary aim of this meta-analysis and systematic review was to investigate the effects of integrative neuromuscular training on athletes’ performance in jumping, sprinting, agility, and balance. The risk of bias was assessed using the Cochrane Risk of Bias (RoB) tool across the 19 included studies. Four studies were rated as high risk of bias [18,31,37,42], eight as moderate [21,22,26,27,29,30,34,39], and seven as low [25,26,28,32,33,35,38]. Overall, the findings from this meta-analysis indicate that INT is effective in improving athletic performance in multiple motor domains.

### 4.1. Effect of Jump Performance

The results of this meta-analysis indicate that integrative neuromuscular training (INT) exerts a statistically significant positive effect on jump performance (SMD = 0.26, 95% CI [0.15, 0.37], I^2^ = 75%, *p* < 0.05). Sensitivity analysis using the leave-one-out method identified the study by Hammami et al. [21] as a potential source of heterogeneity. The elevated heterogeneity may be attributed to the inclusion of multiple components in their INT protocol—such as balance, strength, and power training—combined with sport-specific soccer exercises. Compared to other interventions, the integration of sport-specific elements likely amplified the training effects. Moreover, participants in that study exhibited a higher baseline level of fitness and training adaptation, which may have contributed to the markedly elevated effect size. Hence, the inclusion of this study may have substantially increased overall heterogeneity.

Although the subgroup analysis by age did not reveal statistically significant differences, the results suggested that athletes aged ≥15 showed greater improvements in jump performance compared to those under 15. From a physiological perspective, individuals aged ≥15 are generally in late puberty or early adulthood, with significantly higher levels of androgens and more mature muscular development [43,44]. These factors contribute to greater potential for gains in power and strength, which may explain the more pronounced improvements in jump performance. In contrast, no clear improvements were observed in the <15 group, likely due to the fact that prepubescent individuals have underdeveloped neuromuscular systems, and strength gains are primarily driven by neural adaptations rather than muscle hypertrophy [45].

Subgroup analysis by gender revealed that INT had a significant positive effect on jump performance in male athletes, whereas no significant effect was observed in females. This may be attributed to marked differences in hormonal levels, body composition, and neuromuscular function between males and females. Notably, during puberty, the surge in testosterone in males leads to substantial increases in muscle mass and maximal strength [43,44], giving them a natural advantage in explosive tasks such as jumping and sprinting.

The majority of included studies supported the efficacy of INT in enhancing jump performance. For instance, Bonato et al. [37] employed the countermovement jump (CMJ) as an evaluation metric and reported significant improvements in jump height following INT, indicating enhanced explosive power output. This finding was further corroborated by Mainer-Pardos et al. [23], who suggested that INT enhances lower-limb power, thereby improving both jump and sprint performance. Xiong et al. [22] emphasized that jump capacity is closely related to muscular strength and power. Their training protocol, incorporating both multi-joint (e.g., squats) and single-joint strengthening (e.g., prone leg curls), was designed to improve major lower-limb muscle strength and intermuscular coordination, ultimately contributing to better jump performance.

From a physiological perspective, Bonato et al. [37] proposed that INT improves jump performance by stimulating the muscle–tendon–neural system across multiple dimensions. Specifically, it enhances the stretch-shortening cycle (SSC) by promoting energy storage during the eccentric phase and energy release during the concentric phase, thereby increasing kinetic energy conversion efficiency in explosive actions. Previous studies have further suggested that the principal mechanisms underlying INT’s effectiveness in jump performance enhancement involve improved neuromuscular function and optimized force transmission along the kinetic chain [46,47,48]. Through enhanced neuromuscular coordination, INT refines intermuscular synergy and the timing of muscle activation, enabling athletes to generate force more efficiently during vertical jumps and thereby achieve greater jump heights [49]. With continued neural adaptations, improvements in motor control efficiency and movement stability are also observed, reinforcing the consistency and effectiveness of jump performance over time [50,51].

### 4.2. Effect of Sprint Performance

The results of the present meta-analysis align with previous findings, indicating that integrative neuromuscular training (INT) significantly improves sprint performance in athletes [27,52]. Sensitivity analysis using a leave-one-out approach identified the study by Hammami et al. [21] as a major contributor to heterogeneity. Although the intervention in that study involved only 15 min per session, twice per week, its integration with sport-specific soccer training likely produced synergistic effects that enhanced lower-limb explosive strength. This may explain the elevated effect size and substantial contribution to the observed heterogeneity.

Although males inherently have an advantage in explosive performance, including sprinting and jumping, subgroup analysis showed that females exhibited greater improvements in sprint performance following INT intervention.

Among the 13 studies included in this analysis, INT demonstrated a moderate-to-large positive effect on sprint performance (SMD = −0.76, 95% CI [−0.93, −0.58], *p* < 0.00001, I^2^ = 76%). However, the overall effect was accompanied by considerable heterogeneity across studies. Some studies failed to detect significant improvements, which may be attributed to factors such as participant characteristics or suboptimal training parameters. For instance, Xiong et al. [22] reported a lack of significant effects in elite table tennis athletes, possibly due to a ceiling effect or the insufficient inclusion of speed-specific components in the INT protocol. Similarly, Williams et al. [25] observed that low-intensity, short-term INT interventions did not yield substantial improvements in sprint capacity among adolescent basketball players, highlighting the importance of training dosage and intervention duration.

The enhancement of sprint performance through INT is believed to be closely associated with changes in stride length, stride frequency, and ground reaction force (GRF) [53]. In particular, stride frequency is influenced by muscle stiffness, pre-activation, and stretch reflex mechanisms, while stride length is determined by the peak GRF and motor unit recruitment capacity [54]. Neurological adaptations play a pivotal role in mediating these physiological factors. Specifically, the modulation of muscle stiffness, the timing and magnitude of muscle pre-activation, the sensitivity of stretch reflexes, and the recruitment of motor units are all tightly regulated by the central nervous system [48]. INT incorporates a variety of training modalities, including plyometric exercises, resistance training, speed and agility drills, and proprioceptive training. These multimodal stimuli promote neurophysiological adaptations by enhancing neuromuscular activation (i.e., motor unit recruitment and discharge frequency) and improving the stiffness of key muscle-tendon structures such as the gastrocnemius–Achilles tendon complex [4,47,55].Such adaptations contribute directly to increases in the rate of force development (RFD) and neuromuscular excitability, both of which are critical for sprint performance [56].

### 4.3. Effect of Agility Performance

The present meta-analysis demonstrated a statistically significant positive effect of integrative neuromuscular training (INT) on agility performance (SMD = –0.72, 95% CI [–1.23, –0.21], *p* < 0.006, I^2^ = 74%). However, substantial heterogeneity was observed across the included studies. Sensitivity analysis revealed that the study by Arede et al. [27] was a primary contributor to this heterogeneity. This may be attributed to its comparison between INT and FIFA 11+, a program that functions more as a standardized warm-up routine than as high-intensity neuromuscular training. Additionally, the study’s participants were approximately 11 years old, with relatively low training backgrounds, making their neuromuscular systems more developmentally variable and less predictable in terms of training adaptation.

In the gender-based subgroup analysis, the greater improvements observed in females may be explained by their generally weaker knee stability, muscle balance, and neuromuscular coordination [57,58]. Therefore, the balance, agility, and core control components of INT may more effectively address these deficiencies, resulting in greater performance gains.

Subgroup analysis based on age also indicated more substantial improvements in agility performance among participants aged ≥15. This may be attributed to physiological changes such as increased hormone levels and enhanced neural maturity. Elevated hormone levels promote muscle mass development and structural adaptations of the motor system [59], which in turn improve joint control, acceleration–deceleration capacity, and directional change responsiveness. As strength levels are strongly associated with agility and change-of-direction ability [60], post-pubertal individuals may exhibit greater neuromuscular adaptability in response to INT, which helps explain the more significant improvements in agility observed in this age group.

Several studies have confirmed that INT can significantly improve agility performance [52,61], but the observed heterogeneity may stem from inconsistencies in intervention duration, intensity, and participants’ athletic level. For example, Fernandez-Fernandez et al. [31] implemented a short-duration, low-intensity INT program that failed to enhance agility, suggesting that insufficient training stimulus may not elicit adequate neuromuscular adaptations. Similarly, Gee et al. [35] found that badminton, squash, and soccer players did not show significant improvements in Illinois agility test performance following INT. One explanation is that these sports inherently involve frequent directional changes and rapid starts and stops, which may reduce sensitivity to additional neuromuscular stimuli, resulting in diminishing returns [62]. Despite the high heterogeneity, existing studies have highlighted that INT protocols incorporating change-of-direction (COD) drills, rapid acceleration–deceleration, and multidirectional jump training can elicit meaningful neuromuscular adaptations associated with agility enhancement. Fernandez-Fernandez et al. [31] reported that multidirectional strength and high-velocity explosive training improved coordination of lower-limb musculature and stride frequency control, thereby enhancing force transmission through the kinetic chain. Arede et al. [27] also emphasized that long-term implementation of multidirectional jumping and COD routines contributed to greater neuromuscular activation and control of the involved muscle groups, which is essential for agility development.

The efficacy of INT in improving agility performance is underpinned by various physiological mechanisms, including the stretch-shortening cycle (SSC), proprioception, and balance training adaptations [32,63,64]. SSC plays a central role in COD actions by enabling rapid eccentric–concentric transitions that increase power output and movement efficiency. INT has also been shown to enhance motor unit recruitment, firing frequency, and synchronization, all of which contribute to improved neuromuscular control [20,56]. Agility improvements have also been linked to enhanced proprioception [57], which provides the central nervous system with critical information about body position and movement. Proprioceptive acuity allows athletes to respond rapidly and coordinate complex motor actions effectively during dynamic movements [65]. Consequently, well-developed proprioception is vital for precise postural awareness and movement control, both of which are foundational to agility performance.

### 4.4. Effect of Balance Performance

The results of the present meta-analysis demonstrated a statistically significant positive effect of integrative neuromuscular training (INT) on balance performance (SMD = 0.18, 95% CI [0.09, 0.27], *p* < 0.05; I^2^ = 83%). Substantial heterogeneity was detected among the included studies. Sensitivity analysis identified the study by Zhang et al. [33] as the primary source of heterogeneity. In that study, the participants were approximately 20 years old and were competitive ballroom dancers with over 13 years of training experience. Their advanced baseline postural control and proprioceptive acuity were likely superior to those of participants in other included studies. Moreover, the intervention duration (10 weeks, three sessions per week, 60 min per session) was considerably longer and more intensive than in other trials. The training content emphasized postural control and core stability, and the outcome measures (e.g., the Y-balance test (YBT) and the modified balance error scoring system (BESS)) were highly sensitive to detecting subtle improvements. These factors collectively contributed to an unusually large effect size, which deviated markedly from the overall trend and likely accounted for the high heterogeneity observed.

Gender-specific subgroup analysis indicated that female participants showed significantly greater improvements in balance performance than males following INT intervention. According to Pletcher et al. [66], adolescent females demonstrate superior static balance compared to their male peers and generally possess greater flexibility. These inherent advantages may enable females to derive greater benefits from the proprioceptive and balance elements of INT.

Multiple studies have consistently reported that INT is effective in improving dynamic postural control, lower-limb stability, and proprioceptive function, thereby playing a critical role in both injury prevention and athletic performance enhancement. Bonato et al. [37] found that INT significantly improved performance on the YBT. The underlying mechanisms may involve both peripheral and central adaptations, including enhanced joint position sense, improved proprioceptive feedback, and better anticipatory muscle activation. Xiong et al. [22] also highlighted that the INT protocol, which incorporated core and lower-extremity strength training components, significantly enhanced lower-limb strength—particularly hamstring development in female athletes—which is crucial for knee joint stabilization. Zech et al. [34] further noted that the effectiveness of INT may vary depending on the balance assessment tool used. For instance, the BESS emphasizes sensory-restricted conditions (e.g., visual occlusion, unstable surfaces, etc.), which may make it more sensitive to central nervous system adaptations. It has been hypothesized that the balance improvements observed with INT are predominantly attributed to central neuromuscular adaptations rather than solely peripheral mechanisms.

## 5. Conclusions

The findings of this study indicate that integrative neuromuscular training (INT) can significantly improve athletes’ performance in sprinting, jumping, agility, and balance. Analysis of the included intervention protocols suggests that improvements in each performance domain depend on specific training components. High heterogeneity observed across studies may be attributed to the complexity of training protocols, participants’ training backgrounds, and the sensitivity of outcome measurement tools.

Subgroup analyses further revealed that improvements in jumping performance were more pronounced under conditions involving intervention durations of ≤8 weeks and training frequencies of three sessions per week. For sprinting performance, greater improvements were observed in female athletes. Additionally, interventions conducted more than three times per week and lasting less than 30 min per session yielded more favorable outcomes, particularly when the intervention period did not exceed eight weeks.

## Figures and Tables

**Table 1:** PICO search strategy elements.

PICOS Element	Description	Search Terms
P (Population)	Athletes (professional or amateur) aged ≥11 years, with ≥12 months of training and training ≥ 1 time/week	“athlete*, sport*”,“physical training”
I (Intervention)	Integrative neuromuscular training or neuromuscular training	“Integrative neuromuscular training”, “neuromuscular training”, INT, NMT
C (Comparison)	Conventional training, other types of training, or no intervention	control*, standard training, “no intervention”, placebo
O (Outcome)	Physical performance indicators (e.g., speed, agility, and balance)	performance, speed, agility, balance, “athletic performance”
S (Study design)	Randomized controlled trials with a complete intervention protocol	“Randomized controlled trial”, RCT

Abbreviations: *:Truncation symbol used in database searches to retrieve all terms with the same root.

**Table 2:** Characteries of all studies included.

Authors	Sample Size (M/F, E/C)	Age (Years, Mean ± SD)	Type of Athletes	Time/Week	Training Duration	Performance Test	INT’s Components
Williams 2023 [25]	22 (Mixed, 11/11)	11.4 ± 0.67	Basketball	1	8 weeks	10-M ST, CMJ	Functional Movement Training, Dynamic Stability, Coordination Training, Strength Training, Speed/Agility
Moliner 2023 [26]	38 (0/38, 18/20)	-	Football	3	10 weeks	HJ, CMJ, 40-M ST, COD	Mobility, Stability, Strength, Core Stability, Agility
Pardos 2024 [23]	12 (12/0, 6/6)	13.4 ± 0.36	Tennis	2	10 weeks	HJ, CMJ, DJ, COD, 20-M ST, MBT	Dynamic Stability, Strength, Plyometric, Coordination, Regeneration
Arede 2022 [27]	30 (30/0, 15/15)	11.2 ± 0.70	Football	2	6 weeks	20-M ST, COD	Dynamic Stability, Strength, Plyometric, Coordination, Regeneration
FIlIpA 2010 [28]	20 (0/20, 13/7)	15 ± 1.20	Football		8 weeks	SEBT	Plyometric, Strength, Dynamic Stability
Italo 2023 [29]	37 (37/0, 17/20)	14.31 ± 0.64	Football	2	8 weeks	HT, SHT, CMJ, 10/20-M ST	Strength and Hold, Balance and Strength, Balance and Stability
Emirzeoglu 2021 [30]	32(32/0, 16/16)	15–20	Football	-	-	SEBT	Dynamic Stability, Agility Coordination, Core Control
Fernandez 2020 [31]	29 (29/0, 14/15)	15.09 ± 6.16	Tennis	3	8 weeks	20M-ST, COD, CMJ	Mobility, Core and Trunk Control, Plyometric
Michailidis 2023 [32]	31 (Mixed, 14/17)	14.3 ± 0.60	Football	2	5 weeks	10/20-M ST, SJ, CMJ, COD, IAT	Strength, Plyometric, Core stability, Agility
Zhang 2021 [33]	42 (21/21, 22/20)	20.13 ± 1.79	Ballroom dancing	3	10 weeks	Modified-BESS, YBT	Balance
Zech 2014 [34]	30 (30/0, 15/15)	14.9 ± 3.00	Hockey	2	10 weeks	SEBT, BESS, TTS	Core Stability, Strength, Balance, Plyometrics
Gee 2020 [35]	20 (0/20, 10/10)	22.3 ± 2.0	Gymnastics	2	8 weeks	20-M ST, CMJ, IAT, QASLS	Plyometrics, Core Stability, Strength, Agility, Regeneration
Xiong 2022 [22]	24 (0/24, 12/12)	23.2 ± 2.25	Table tennis	4	8 weeks	VJ, YBT, 30M-ST	-
Steffen 2013 [36]	158 (0/158, 78/80)	13–18	Football	2	4–5 months	Single-leg eyes-closed balance, SEBT, SLTH	Agility, Plyometrics, Functional Movement Training, Strength, Balance
Fernandez 2018 [18]	16 (Mixed, 8/8)	12.9 ± 0.40	-	-	5 weeks	5/10/20M-ST, CMJ, SV, MBT, COD	Plyometrics, Agility,
Bonato 2018 [37]	160 (0/160, 86/74)	20 ± 2.00	-	4	8 months	CMJ, YBT	Agility, Strength, Plyometrics, Agility, Speed
Benis 2016 [38]	28 (0/28, 14/14)	20 ± 2.00	Basketball	2	8 weeks	YBT	Core Stability, Strength, Balance, Plyometrics, Agility
Hammami 2023 [21]	24 (24/0, 12/12)	15.5 ± 0.70	Football	2	8 weeks	YBT, FJT, CMJ, single-leg hop, 30M-ST, COD with ball	Balance, Strength, Agility/Speed
Ayala 2017 [39]	30 (Mixed, 10/20)	16.8 ± 0.70	Football	4	4 weeks	Single-leg hop limb, YBT, 10/20M-ST, Agility, VDJ	Strength, Balance, Muscle Control, Core Stability, Speed

Abbreviations: M: male; F: female; E: experiment group; C: control group; SD: standard deviation; SEBT: star excursion balance test, MBT: medicine ball throw test, VJ: vertical-jump test, FJT: five jump tests, SLTH: single-leg triple hop, SJ: squat jump, CMJ: countermovement jump, DJ: deep jump, HJ = horizontal jump test, COD = 505 change of direction test, YBT = Y-balance test, BESS = balance error scoring system, TTS = time to stabilization, QASLS = qualitative analysis of a single-leg squat, IAT = Illinois agility test, SV = serve velocity test, ST = sprint test.

**Table 3:** Subgroup of jump performance.

Subgroup	Number of Studies	Effect Size	95% CI	*p*-Value	I2
Age					
<15 years old	5	0.91	(0.54, 1.27)	0.543	0
≥15 years old	17	0.62	(0.41, 0.83)	0.01	50%
Mixed	6	−0.51	(−0.78, −0.24)	0.002	73.90%
Sex					
Male	17	0.7	(0.49, 0.92)	0.243	18%
Female	10	0.03	(−0.14, 0.19)	0.001	85.90%
Mixed	2	0.03	(−0.45, 0.50)	0.96	0
Intervention Time					
<30 min	2	−0.12	(−0.32, 0.08)	0.001	75.60%
≥30 min	16	0.87	(0.64, 1.10)	0.094	33.50%
Intervention Duration					
≤8 weeks	13	0.73	(0.52, 0.94)	0.002	61.60%
>8 weeks	16	0.01	(−0.15, 0.17)	0.001	67%
Intervention Frequency					
2 days/weeks	19	0.47	(0.31, 0.62)	0.001	56.90%
≥3 days/weeks	9	−0.15	(−0.38, 0.08)	0.001	82.10%

**Table 4:** Subgroup of sprint performance.

Subgroup	Number of Studies	Effect Size	95% CI	*p*-Value	I^2^
Age					
<15 years old	11	−0.37	(−0.62, −0.13)	<0.001	76.40%
≥15 years old	7	−0.62	(−0.93, −0.31)	0.754	0
Sex					
Male	8	−0.36	(−0.63, −0.09)	0.002	69.20%
Female	6	−1.57	(−1.90, −1.24)	0.003	72%
					
Intervention Time					
<30 min	10	−1.07	(−1.32, −0.83)	0.001	78.70%
≥30 min	12	−0.44	(−0.69, −0.19)	<0.001	72.40%
Intervention Duration					
<8 weeks	17	−0.48	(−0.67, −0.28)	<0.001	66%
≥8 weeks	5	−0.18	(−2.17, −1.42)	0.041	59.80%
Intervention Frequency					
2 days/weeks	9	−0.26	(−0.52, −0.01)	0.006	62.70%
≥3 days/weeks	12	−1.33	(−1.58, −1.08)	0.001	65.90%

**Table 5:** Subgroup of agility performance.

Subgroup	Number of Studies	Effect Size	95% CI	*p*-Value	I^2^
Age					
≤15 years old	6	0.25	(−0.12, 0.63)	<0.001	85.90%
>15 years old	5	−0.73	(−1.11, −0.35)	<0.001	85.30%
Sex					
Male	7	0.06	(−0.28, 0.39)	<0.001	89.40%
Female	3	−0.43	(−0.84,−0.02)	0.336	8.4%
Intervention Time					
<30 min	6	−0.56	(−0.86, −0.25)	<0.001	81.60%
≥30 min	7	0.17	(−0.18, −0.53)	<0.001	84.70%
Intervention Duration					
≤8 weeks	9	−0.27	(−0.55, 0.01)	<0.001	89.30%
≥8 weeks	4	−0.19	(−0.58, 0.20)	0.857	0
Intervention Frequency					
2 days/weeks	6	−0.44	(−0.74, −0.13)	0.01	66.80%
≥3 days/weeks	7	0	(−0.34, 0.35)	0.001	89.60%

**Table 6:** Subgroup of Balance performance.

Subgroup	Number of Studies	Effect Size	95% CI	*p*-Value	I^2^
Sex					
Male	14	0.05	(−0.14, 0.25)	<0.001	66.80%
Female	22	0.25	(0.15, 0.34)	<0.001	84.90%
mixed	4	0.75	(0.36, 1.15)	0.593	0
Intervention Time					
<30 min	13	0.77	(0.54, 1.00)	<0.001	88.10%
≥30 min	19	0.41	(0.27, 0.56)	0.002	55.30%
Intervention Duration					
≤8 weeks	17	1.36	(1.44, 1.59)	<0.001	71%
>8 weeks	16	0.07	(−0.02, 0.16)	0.367	7.5%
Intervention Frequency					
2 days/weeks	27	0.24	(0.15, 0.32)	<0.001	83.70%
≥3 days/weeks	3	0.75	(0.36, 1.15)	0.0005	95.90%
